# Driving chronicity in rheumatoid arthritis: perpetuating role of myeloid cells

**DOI:** 10.1111/cei.13098

**Published:** 2018-02-02

**Authors:** S. Alivernini, B. Tolusso, G. Ferraccioli, E. Gremese, M. Kurowska‐Stolarska, I. B. McInnes

**Affiliations:** ^1^ Institute of Rheumatology, Fondazione Policlinico Universitario A. Gemelli – Catholic University of the Sacred Heart Rome Italy; ^2^ Institute of Infection, Immunity and Inflammation, University of Glasgow; ^3^ Rheumatoid Arthritis Pathogenesis Centre of Excellence (RACE) Glasgow UK

**Keywords:** arthritis (including rheumatoid arthritis), cytokines, inflammation, macrophage

## Abstract

Acute inflammation is a complex and tightly regulated homeostatic process that includes leucocyte migration from the vasculature into tissues to eliminate the pathogen/injury, followed by a pro‐resolving response promoting tissue repair. However, if inflammation is uncontrolled as in chronic diseases such as rheumatoid arthritis (RA), it leads to tissue damage and disability. Synovial tissue inflammation in RA patients is maintained by sustained activation of multiple inflammatory positive‐feedback regulatory pathways in a variety of cells, including myeloid cells. In this review, we will highlight recent evidence uncovering biological mechanisms contributing to the aberrant activation of myeloid cells that contributes to perpetuation of inflammation in RA, and discuss emerging data on anti‐inflammatory mediators contributing to sustained remission that may inform a novel category of therapeutic targets.

## Introduction

Rheumatoid arthritis (RA) develops from breach‐of‐tolerance to self‐antigens and the generation of autoantibodies to post‐translationally modified self‐proteins, e.g. via citrullination and carbamylation, to create anti‐modified protein autoantibodies (AMPA), that probably initiate inflammation and bone damage. RA pathogenesis comprises genetic predisposition (∼100 alleles mainly with immune function, e.g. human leucocyte antigen D‐related 4 (HLA‐DR4): DRB1*0401) 1, and epigenetic changes including DNA/histone modification and microRNA deregulation. Environmental factors such as cigarette smoking associate with disease perhaps by enhancing post‐translational citrullination forming new epitopes that bind preferentially to HLA‐DR molecules (e.g. DRB1*0401) compared with the native form. This, acting in concert with less‐understood stimuli such as infection, exposure to silica particles or an altered microbiota, lead to dendritic cell (DC)‐orchestrated (or potentially B cell) activation of citrullinated peptide‐specific T helper type 1 (Th1)‐producing IFN‐γ and Th17‐producing IL‐17 and granulocyte–macrophage colony‐stimulating factor (GM‐CSF) and follicular T lymphocytes, which facilitate production of autoantibodies [e.g. anti–citrullinated protein antibody (ACPA)].

Initiation of autoimmunity precedes onset of joint inflammation and progression to joint degradation, which additionally involves epigenetically altered synovial fibroblasts; the main producers of interleukin (IL)‐6 and of metalloproteinases that degrade cartilage and monocyte‐derived inflammatory macrophages; and the main producers of pathogenic cytokines, notably tumour necrosis factor (TNF)‐α 2, 3. The factors that initiate the localized inflammation in the joint remain poorly understood. It is proposed that joint injury or transient infection can activate the vasculature and allow access of autoantibodies. These form immune complexes with joint tissue antigens and activate stromal cells, resident macrophages, mast cells and osteoclasts to release vasoactive mediators and chemokines recruiting leucocytes and exacerbating the inflammation 2, 3. For example, monocytes infiltrating the synovium differentiate locally to macrophages and are the main source of TNF, IL‐1β, IL‐6 and GM‐CSF 4. Alternate initiating models propose ACPA‐binding osteoclasts and inducing local IL‐8 production and neutrophil recruitment and this inflammation onset 5. DCs, mainly the CD1c^+^ subpopulation, contribute to the proinflammatory cytokine milieu in the synovium and activate autoreactive T cells 6.

The majority of current RA treatments target either these inflammatory mediators or their critical signalling pathways, e.g. TNF (soluble receptor or blocking antibodies), IL‐6R (blocking antibody), Janus kinases (small molecule inhibitors) or target adaptive immunity, e.g. B cells (anti‐CD20 antibody) or block the interaction between antigen‐presenting cells and T cells [cytotoxic T lymphocyte antigen‐4 (CTLA‐4)]. These treatments retard disease progression, but do not reverse underlying autoimmunity and do not cure the disease. Regardless of treatment, 30–50% of patients exhibit an inadequate response, and among those who respond, 50% relapse within weeks after treatment cessation 2, highlighting the need for novel treatment strategies. Obvious targets based on murine model predictions have, unexpectedly, failed. For example, therapies targeting either IL‐17 or the common subunit of IL‐12/IL‐23 (cytokines that drive Th1 and Th17, respectively) did not improve RA disease activity 7. Thus, a better understanding of the pathogenesis of RA is required to prosper new therapies. This review will describe recent evidence uncovering biological mechanisms contributing to the aberrant activation of myeloid cells that leads to perpetuation of inflammation and chronicity in RA. We will review recent evidence for inflammatory mediators that are novel therapeutic targets, e.g. GM‐CSF, and describe novel functions of well‐described inflammatory mediators, e.g. TNF, that could explain their contribution to the chronicity of RA. We will also discuss emerging data describing the role of anti‐inflammatory mediators in sustained remission in RA patients, and how these may lead to the development of new therapeutics.

## Synovial myeloid cells are key perpetuators of inflammation

Inflammation is a complex homeostatic process that includes leucocyte migration from the vasculature into tissues. This is followed by activation of negative‐feedback mechanisms that should attenuate inflammation to limit collateral damage and initiate repair. The success of therapies against myeloid cell derived mediators and *ex‐vivo* studies suggest that synovial inflammation is maintained by sustained activation of multiple inflammatory positive‐feedback loops in myeloid cells 8, 9 fuelled by epigenetically modified tissue‐resident cells 10 and effector pathways of autoimmunity 5.

### Macrophages

Animal models show that most healthy tissues contain tissue‐resident macrophages that originate from prenatal precursors and are essential in maintaining tissue homeostasis, e.g. by removing apoptotic cells. In response to infection/injury, a different tissue macrophage population differentiates from infiltrating monocytes and these mount an inflammatory response 11. Experimental data show that mouse synovium contains a tissue‐resident macrophage population that is crucial in limiting inflammatory responses in the synovium, while monocyte [both lymphocyte antigen 6 complex, locus C1 (Ly6C)^+^ and Ly6C^–^]‐derived macrophages drive the inflammatory response and the severity of experimental arthritis 12, 13. Transcriptomic data suggest that these two subpopulations of macrophages respond differently to ‘on‐demand’ signals derived from a changing tissue environment, which can polarize macrophages on the spectrum from proinflammatory (M1 activation spectrum) to anti‐inflammatory and repair (M2 activation spectrum) 12. This has been confirmed elegantly in a recent paper showing that tissue‐resident macrophages (e.g. in the peritoneum, plural cavity or lung) have a Krüppel‐like factor 2 (KLF2)/KLF4‐driven transcriptomic programme that facilitates apoptotic cell uptake while negatively regulating proinflammatory Toll‐like receptor (TLR) signalling 14. Similarly, in mouse models of arthritis, tissue‐resident macrophages and monocyte‐derived macrophages exhibit distinct transcriptomic profiles at the peak of inflammation. Monocyte‐derived macrophages showed the M1‐type phenotype with high levels of proinflammatory mediators, e.g. IL‐1β, IL‐12, CD80 and CD86, compared to resident macrophages, while the latter up‐regulated efferocytosis receptors [e.g. MER proto‐oncogene, tyrosine kinase (MerTK), CD36, CD163]. During the resolution of experimental arthritis, there is a phenotypical change of monocyte‐derived macrophages from a pro‐ to anti‐inflammatory (M2 spectrum) that is induced by an as‐yet‐unidentified signal 12.

**Figure 1:**
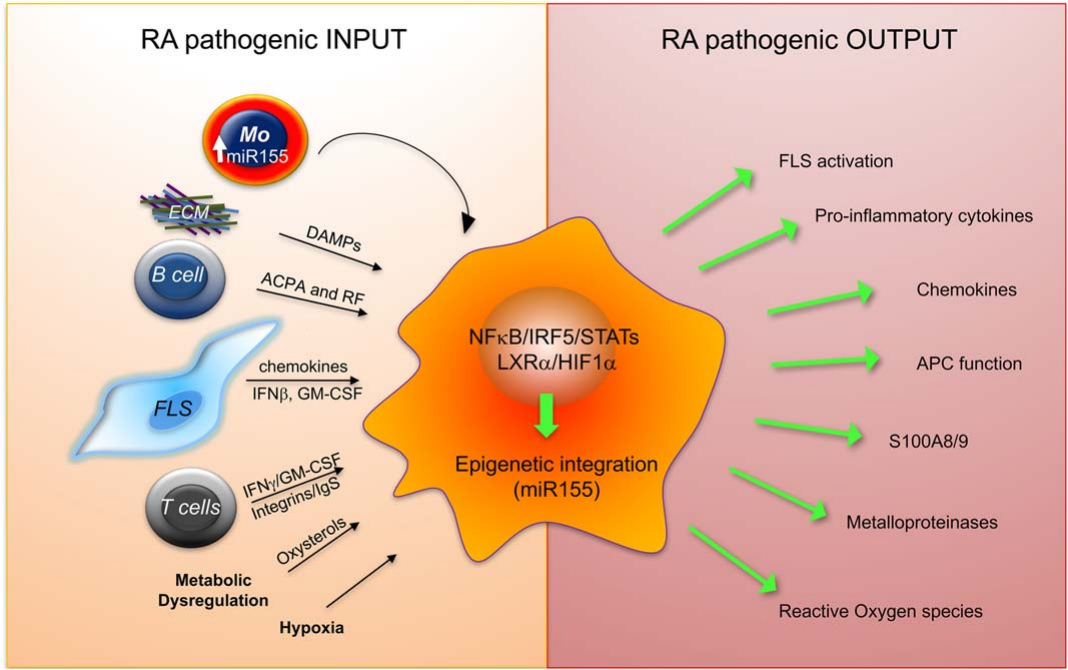
Mechanisms of macrophage cell activation promoting chronic inflammation in rheumatoid arthritis. Synovial macrophages differentiate from epigenetically reprogrammed monocytes that migrate from the circulation into synovial fluid and tissue. Synovial damage‐associated molecular patterns (DAMPs) (e.g. fibronectin) binding to Toll‐like receptors (TLRs), anti‐citrullinated protein antibody (ACPA) or rheumatoid factor binding to Fc‐gamma receptor (FcyR), oxysterols, hypoxia and direct integrin‐mediated and cytokine‐mediated crosstalk with T cells and fibroblast‐like synoviocytes (FLSs) promote synovial macrophage proinflammatory activation. This response is co‐ordinated by several transcription factors and integrated by epigenetic regulators, e.g. microRNA155 that inhibits a negative‐regulator of inflammation Src homology 2‐containing inositol phosphatase‐1 (SHIP1); thus, maintains synovial macrophages chronic activation. ECM = extracellular matrix proteins; GM‐CSF = granulocyte‐macrophage colony‐stimulating factor; HIF1α = hypoxia‐inducible factor 1‐alpha; IgS = Ig‐superfamily; IRF5 = interferon regulatory factor 5; LXRα = Liver X Receptor alpha; miR = microRNA; MMPs = metalloproteinases; NF‐κB = nuclear factor kappa B; RF: rheumatoid factor; STAT = signal transducer and activator of transcription. This figure presents a novel development of concepts that were presented in our previous review 75.

In human healthy joints, the synovial lining layer expresses tissue‐resident macrophages that contain phagosomes and express the scavenger receptor CD163, suggesting that, similar to their mouse counterparts, they are strongly phagocytic 15, 16. They also express the major histocompatibility complex (MHC)‐II, IL‐1R‐antagonist, the inhibitor of bone degradation, osteoprotegerin (OPG) 15, 17, 18, but they lack proinflammatory cytokines (e.g. TNF and IL‐1β) and the bone resorption‐inducing cytokine, receptor activator of nuclear factor kappa‐Β ligand (RANKL) 18, suggesting a joint‐protective function during inflammation and damage. During RA development, driven by an influx of blood monocytes 19, the number of macrophages increases, particularly in the sublining layer, and they polarize into a proinflammatory phenotype upon activation by local signals. The hypertrophic synovium creates a hypoxic environment 20, and recruited macrophages adapt to the low oxygen levels by up‐regulating hypoxia‐inducible transcription factor 1 (HIF‐1α) that mediates a switch in their energy metabolism from an oxidative phosphorylation to anaerobic glycolysis, and supports their proinflammatory activation 21, 22. Myeloid‐specific deletion of HIF‐1α reduces joint swelling and inflammatory activity in a murine arthritis model 23. ACPA and citrullinated fibrinogen complex in the synovium can induce TNF production via synergistic binding to FcγΡ and TLR‐4 24. Oxidized oxysterols, enriched in RA synovial fluid, can bind to the transcription factor liver X receptor (LXRα) in synovial macrophages and enhance damage‐associated molecular patterns (DAMPs)‐induced TNF, IL‐6, IL‐1β and granulocyte–macrophage colony‐stimulating factor (G‐CSF) production 25. Memory T cells, recruited and expanded in synovial fluid by cytokines (e.g. IL‐15, IL‐6 and TNF), trigger substantial production of TNF by macrophages upon direct integrin‐mediated contact 26, 27, while IFN‐γ released by citrullinated‐peptide autoreactive T cells 28 can enhance the ability of macrophages to present antigen and thereby their potential to activate autoreactive memory T cells. Activated by the local stimuli, RA synovial macrophages produce predominantly chemokine (C‐X‐C motif) ligand (CXCL)4 and CXCL7 (chemokines recruiting neutrophils and blood monocytes), particularly in early RA 29, and TNF 30 and other proinflammatory cytokines (e.g. IL‐1β 31 and IL‐6 32) and alarmins S100A8/9 33 throughout disease progression. Their proinflammatory activation is co‐regulated by nuclear factor kappa B (NF‐κB), interferon regulatory factor 5 (IRF5), signal transducer and activator of transcriptions (STATs), LXRα and HIF‐1α transcription factors at the transcriptional level 20, 25, 34, and by microRNA155 and microRNA146 at the post‐transcriptional level 35, 36. In multi‐cellular organisms, miRNAs are small non‐coding RNAs that repress their target mRNAs by binding to complementary sequences at the 3' untranslated regions 37. MicroRNA155 was identified as a key positive regulator of the inflammatory response that is counterbalanced by the action of microRNA146 in myeloid cells 38, 39. MicroRNA155 expression is increased significantly in RA synovial tissue macrophages and in synovial fluid and blood monocytes, compared to non‐inflammatory synovial tissue and to healthy blood monocytes. Expression of microRNA155 in RA monocytes correlates strongly with disease activity 40. The proinflammatory environment in arthritic joints (e.g. DAMPs, TNF, IL‐1β, oxysterols and contact with T cells) triggers and maintains microRNA155 expression in synovial fluid monocytes and in tissue macrophages, and this locks these cells into a chronic proinflammatory state 35, 40, 41, 42. MicroRNA155 represses Src homology 2‐containing inositol phosphatase‐1 (SHIP‐1), which is a negative‐regulator of the phosphoinositide 3‐kinase/Akt pathway, and a negative‐regulator of TLR signalling. Moreover, high expression of microRNA155 leads to the repression of CCR2 in monocytes, and this probably contributes to the retention of synovial monocytes in the synovium 40. Neutralization of microRNA155 in synovial fluid cells abolished their TNF production and de‐repress the expression of SHIP‐1, thus restoring the homeostatic negative‐feedback mechanism limiting inflammation 35 (Fig. 1).

### Dendritic cells

DCs maintain immune tolerance to self‐antigens in healthy tissues. During infection they undergo maturation, induced by ligand binding of pathogen‐associated molecules to cell receptors (e.g. TLR), and this enables them to activate the immune response to promote pathogen elimination. When the infection is cleared, DCs then reinstate homeostasis to prevent further tissue damage and potential for bystander activation of autoreactive T cells. Tolerance and immune homeostasis are mediated by immature and tolerogenic DCs characterized by unique phenotypes. These include low expression of co‐stimulatory molecules (e.g. CD40, CD80, CD86) and the absence of production of proinflammatory mediators (e.g. IL‐12). They express inhibitory receptors [e.g. programmed death ligand 1 (PD‐L1), CD95L] and produce inhibitory mediators preferentially [e.g. IL‐10, TGF‐β, IL‐27, prostaglandin E_2_ (PGE_2_) and indoleamine 2,3‐dioxygenase]. These DC phenotypes prevent activation of autoreactive T cells and down‐regulate adaptive and inflammatory immune responses by several mechanisms, including: by inducing apoptosis or anergy in effector T cells, by expansion of thymus‐derived regulatory T cells (tT_reg_) and by generation of new regulatory T cells (e.g. T_reg_1). Regulatory T cells can limit the activation of antigen‐specific effector T lymphocytes and the activation of proinflammatory macrophages. In addition, they can feedback‐inhibit DC activation, thus restraining the immune response and reinstating homeostasis. DCs are categorized broadly as plasmacytoid DCs or conventional myeloid DCs that differ in location, the molecular signals they recognize and the profile of mediators they produce 43, 44.

CD1c^+^ dendritic cells in humans, and their murine counterparts CD11c^+^CD11b^+^ dendritic cells, are the most abundant subpopulation of myeloid DCs in tissues where their immune‐regulatory immature/tolerogenic phenotype supports regulatory T cells and maintains tissue homeostasis 45, 46, 47. Upon infection they mature, particularly upon TLR‐7/8 ligation, and initiate an immune response to pathogens by activation of Th1 and Th17 lymphocytes 48, 49. Dendritic cells are found in the healthy synovium and localize mainly in the perivascular areas of the sublining layer 15, probably contributing to maintaining tolerance in the joint. Evidence points towards a significant role of CD1c^**+**^ DCs in the onset and progression of RA. ACPA‐positive^(^^**+**)^ healthy individuals, at risk of developing RA, have substantially increased numbers of CD1c^**+**^DCs in the lymph nodes that drain joint tissue, compared with ACPA‐negative^(^^**–)**^ healthy individuals 50. This suggests their potential involvement in the regulation of early breach‐of‐self‐tolerance in preclinical RA. Thereafter, synovial tissue and fluid from patients with active RA are enriched in CD1c^**+**^ DCs that have an activated (mature) phenotype, indicated by increased expression of co‐stimulatory molecules such as CD86, CD80 and CD40 compared to blood cells. They can expand autologous memory Th1/Th17 cells 51, 52, 53 and contribute to synovial inflammation by producing TNF upon TLR‐7/8 activation 54, 55 and chemokines 51. It is postulated that their mature phenotype in the synovium is mediated by thymic stromal lymphopoietin (TSLP) produced locally in the inflamed joint by activated fibroblasts‐like synoviocytes 52.

Commensurate with an increased number of CD1c^+^ in joint draining lymph node (DLN) of individuals at risk of developing RA, circulating CD1c^+^ of patients with early RA are changed epigenetically and show increased expression of microRNA34a, which is sustained in established RA and up‐regulated further in synovial tissue CD1c^+^ cells. This, correlated with decreased expression of its mRNA target *Axl*, a tyrosine kinase receptor that together with other family members, Tyro3 and MerTK, are indispensable in limiting DC activation 56. MicroRNA34a has been identified as an evolutionarily conserved key regulator of innate immunity in Drosophila 57. Recently, it was shown that the microRNA34a/GAS6‐Axl pathway is important for optimal control of the immune response 58. CD1c^+^ DC stimulation by TLRs leading to DC‐driven effector T cell activation is commensurate with down‐regulation of microRNA34a and release of its epigenetic control of Axl in DCs. Axl, upon binding of its ligand GAS6, induces SOCSs (suppressor of cytokine signalling) and terminates DC proinflammatory cytokine production and DC‐driven T cell activation. With age, both Axl deficient(^–/–^) and GAS6^–/–^ mice develop an autoimmune phenotype spontaneously. Axl^–/–^ mice have DCs with an activated phenotype, and develop increasing serum titres of autoantibodies resembling the early breach‐of‐tolerance to self‐antigen characteristic of preclinical phase RA 59, while GAS6^–/–^ mice develop a spontaneous increase in effector Th17 over T_regs_ that leads to gut inflammation 60. The production of proinflammatory cytokines and expression of MHC class II by DCs could be inhibited by gene‐silencing of microRNA34a, and in keeping with this, microRNA34a^**–/–**^ mice do not develop collagen‐induced arthritis, and their DCs have diminished interaction with effector antigen‐specific T cells, reduced production of proinflammatory cytokines and did not support the development of pathogenic Th17 cells. These functions were attributable to the corresponding increased expression of deregulated *Axl* 58. Neutralization of the increase expression levels of microRNA34a in CD1c^+^DCs from RA patients inhibited proinflammatory cytokine production by reinstating the expression of the Axl inhibitory pathway. Consistent with this, van den Brand *et al*. 61 showed that activation of the Axl pathway by GAS6 inhibited experimental arthritis. Thus, optimal activation of the microRNA34/GAS6–Axl pathway is required for DC control of the adaptive immune response, and low expression of Axl in CD1c^+^ DCs in rheumatoid arthritis patients could contribute to the initiation and perpetuation of disease by facilitating the activation of autoreactive T cells upon initial bystander trigger, e.g. infection or tissue damage. This highlights the microRNA34a/GAS6‐Axl pathway for potential therapeutic targeting in RA.

**Figure 2:**
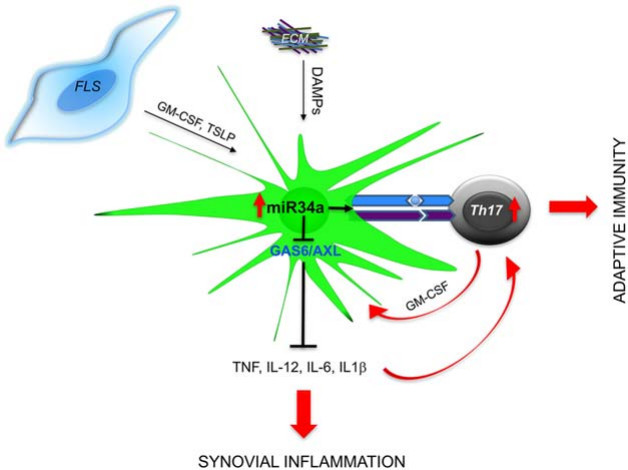
Mechanisms of dendritic cell activation promoting chronic inflammation in and adaptive immunity in rheumatoid arthritis (RA). Local production of GM‐CSF and TSLP (e.g. by FLS) and release of DAMPs activate synovial CD1c^+^ dendritic cells (DCs) to release proinflammatory mediators and to stimulate autoreactive T cells. Synovial CD1c^+^ and their blood precursors express high levels of epigenetic regulator microRNA34a that sustains their proinflammatory and T helper type 17 (Th17) differentiating cytokine production, antigen presentation by major histocompatibility complex (MHC) class II and interaction with T cells. This is, in part, mediated by microRNA34a‐ induced repression of Axl. Axl, upon binding of its ligand GAS6, induces SOCSs (suppressor of cytokine signaling) and terminates DC proinflammatory cytokine production and DC‐driven T cell activation. Thus, low expression of Axl in CD1c^+^DCs in RA patients could contribute to the initiation and perpetuation of disease by facilitating the activation of autoreactive T cells and cytokine production upon initial bystander trigger of dendritic cells (DC), e.g. infection or tissue damage. AXL = AXL‐receptor tyrosine kinase; DAMPs = damage‐associated molecular patterns; ECM = extracellular matrix proteins; FLS = fibroblast‐like synoviocytes; GAS6 = growth arrest‐specific 6; GM‐CSF = granulocyte‐macrophage colony‐stimulating factor; miR = microRNA; TSLP = thymic stromal lymphopoietin.

The results of two recent phase I clinical trials showing safety and efficacy of passive transfer of autologous, *in vitro*‐generated tolerogenic DCs for RA therapy, suggest that targeting DCs to reinstate immune tolerance to citrullinated peptides is a promising therapeutic strategy 62, 63. However, there are challenges, including the reduced likelihood of treatment response in RA patients with long‐standing disease, and the selection of autoantigens for the generation of effective tolerogenic DCs *ex vivo* (Fig. 2).

MicroRNAs offer therapeutic potential either as direct targets or by ‘identifying’ tractable and plausible pathways. Therapeutic targeting of microRNAs has been successful in some cancers, infectious diseases and recently models of tendinopathy 64, 65. Inhibition of microRNA155 with specific anti‐miR‐155 improved the clinical severity of experimental arthritis significantly 66 However, the prospect of non‐cell/tissue‐specific targeting microRNA155 in RA may be unrealistic. MicroRNA155 is key in mounting protective antibody production, regulation of lipid metabolism in the liver and the repair process. MicroRNA155‐deficient mice show reduced germinal centre formation 67, liver steatosis 36 and lung fibrosis 68; thus, systemic targeting of microRNA155 may have detrimental effects. Activation of inhibitors of proinflammatory cell activation that are repressed by microRNA155, e.g. SHIP‐1, by specific activators might be a safer option 69. The SHIP‐1 activator (AQX‐1125) used in clinical trials in chronic obstructive pulmonary disease (COPD) and allergic asthma demonstrated a favourable safety profile and anti‐inflammatory activity (trial number: NCT01954628).

The microRNA34/GAS6–Axl axis may resemble the PDL1/PD1 or CTLA‐4/B7 pathways in the mechanism by which they regulate the balance between immunosuppression (cancer) and immunostimulation (autoimmunity). The recently developed PDL1/PD1 pathway blockade is a highly promising therapy that has elicited anti‐tumour responses and long‐term remissions in a broad spectrum of cancers by activating anti‐cancer‐specific immune response 70. However, these patients not infrequently exhibit side effects that include developing autoimmune disease 71, 72. In contrast to RA, microRNA34a is down‐regulated in stromal cell cancers leading to de‐repression of target anti‐apoptotic proteins and cell‐cycle inhibitors and causing cancer cell expansion 73. The microRNA34amimic MRX34 (Mirna Therapeutics, Austin, TX, USA) is currently being tested in several solid and haematological malignancies 64. Its epigenetic target Axl is de‐repressed in cancer cells and in immune cells of cancer patients and this contributes to immunosuppression and cancer metastasis. This ying–yang role of the microRNA34a/GAS6‐Axl pathway in cancer and autoimmunity suggests that manipulation of this pathway has the potential to rebalance the immune system 74. Thus, therapeutic stimulation of Axl functions in RA DCs to rebalance the immune system could bring clinical improvement in RA, similar to CTLA‐4 therapy, albeit with careful cancer risk factor monitoring.

### Inflammatory mediators

The contribution of macrophage‐ and inflammatory DC‐derived cytokines to RA pathogenesis has been reviewed extensively 4, 7, 9, 75. Briefly, TNF is bioactive as a transmembrane protein as well as a homotrimeric secreted molecule 76. During inflammation, TNF functions by binding two distinct TNF receptors, p55TNFR (TNFRI) and p75TNFR (TNFRII) 77. TNF increases vascular permeability and enhances tissue ingress of cells, immunoglobulin (Ig)G/IgM‐immune complexes and complement 78. TNF synergizes with IL‐1β to induce synovitis by induction of chemokines, cytokines, e.g. IL‐6, and prostaglandins by various cells, causes cartilage destruction by triggering the production of metalloproteinases by synovial fibroblasts and causes bone resorption by increasing the differentiation of osteoclasts 79. Commensurate with this, transgenic over‐expression of TNF triggers the development of chronic synovitis 80. TNF also prevents resolution actively, e.g. by impacting negatively the function of regulatory T cells 81, by inducing forkhead box protein 3 (FoxP3) degradation 82 and by inhibiting the development of TLR tolerance 83. An elegant paper led by Ivaskhiv 83 showed that TNF co‐operates with IFN‐α in preventing the development of tolerance to TLR stimuli. TLR tolerance is a key process in limiting collateral tissue damage during the normal immune response. TNF preconditioning induces tolerance and limits potentially toxic induction of NF‐κB target genes encoding inflammatory molecules, while enabling expression of genes encoding anti‐viral molecules upon LPS stimulation. However, TNF in the presence of IFN‐α reprogrammes the human macrophage epigenome to increase NF‐κB‐driven inflammatory responses to TLR stimulation. Type I interferons potentiate the inflammatory function of TNF by preventing the silencing of genes encoding inflammatory molecules, including IL‐6, IL‐8, CCL4, CCL4 and CCL20. Thus, this cross‐talk between DAMPs (e.g. products of joint damage 84, IFN‐α/β (*e.g*. released upon stimulation with RNA from necrotic joint cells 85 and TNF could contribute to the failure of resolution in the inflammatory RA synovium 83.

Recently, a novel survival role for cell‐intrinsic‐TNF in circulating monocytes was described. Mouse monocytes selectively deficient for TNF or TNF receptors are out‐competed by their wild‐type counterpart due to their decrease survival rate and reduced migration to injured tissues 86. This novel mechanism can contribute to the persistence of inflammatory monocytes and/or macrophages in RA in a TNF‐rich environment. Synovial fluid monocytes from RA patients are resistant to spontaneous apoptosis and to apoptosis mediated by an agonistic anti‐Fas antibody, compared to blood monocytes of healthy donors. This resistance is due to the progressive inhibition of caspace 10 (CASP10) and apoptotic protease activating factor 1 (APAF1) by the increasing levels of microRNA155 87 that is inducible by TNF.

Another cytokine that is essential in the development of RA, as evidenced by the success of the anti‐cytokine therapy, is IL‐6. IL‐6 is produced by various cells, including synovial fibroblasts, macrophages and inflammatory DCs. IL‐6 signals through IL‐6Rα/gp130. IL‐6Rα expression is restricted to leucocytes and a few other cell types, and is functionally active only after combined ligation with gp130. A soluble form of the receptor (s‐IL6Rα) is shed by cells in the circulation and the IL‐6/sIL‐6Rα can dimerize with membrane gp130 present on IL‐6Rα‐negative cells and stimulate these cells. IL‐6 regulates leucocyte recruitment *in vivo* by activating endothelial cells to increase adhesion molecule expression and produce CCL2 88, and promote the transition from neutrophilic to monocytic inflammation by shifting the profile of chemokines produced 89. IL‐6 also regulates T cell infiltration by governing the production of appropriate chemokines, e.g. CXCL10, CCL11, CCL4, CCL17 and the expression of their receptors CCR3, CCR4, CCR5 and CXCR3 on T cells 90. IL‐6 is indispensable for Th17 and follicular T cell differentiation and survival, and for antibody production by B cells 4. In animal models of arthritis, increased IL‐6 is detected from the very early phases of disease consistent with its role in modulating leucocyte recruitment *in vivo* 91. In addition, analysis of blood samples obtained from ACPA^**+**^ individuals at risk of developing RA but without clinical symptoms showed that the concentration of IL‐6 was increased compared to heathy ACPA^–^ subjects 92. An increased expression of an IL‐6‐induced gene signature was found in circulating T cells of early RA 93, while an increased expression of IL‐6 mRNA was found in circulating CD1c^**+**^ DCs which correlated negatively with lower expression of Axl, which is the negative regulator of DC activation 58. These observations suggest the potential involvement of IL‐6 in DC/T cell interaction in early RA.

## Novel therapies targeting myeloid cell relevant anti‐inflammatory mediators

In the synovium, the polarization of monocyte‐derived macrophages and dendritic cells can be shaped by their interaction with synovial CD4^+^ T cells 94 and synovial fibroblasts that are epigenetically reprogrammed to produce a wide range of mediators 95; both cell types produce GM‐CSF that potentiates the proinflammatory activation of macrophages 96, 97 and the differentiation of inflammatory DCs. It was shown recently that GM‐CSF induces metabolic reprogramming of myeloid cells that is critical for an increase production of proinflammatory cytokines following TLR restimulation. GM‐CSF increases the macrophage glycolytic metabolism by a c‐Myc‐dependent mechanism. In addition, GM‐CSF‐primed macrophages have an enhanced activation of the mevalonate pathway due to an increase in expression of enzyme 3‐hydroxy‐3‐methylglutaryl‐CoA reductase (HMGCR) 98 and due indirectly to substrate flux from increased glycolysis. Inhibition of the mevalonate pathway by simvastatin completely abolished GM‐CSF priming. In addition, GM‐CSF drives CCL17 production by acting through an IRF4‐dependent pathway in human monocytes. In murine models of arthritis, GM‐CSF up‐regulated IRF4 and thus CCL17 production by enhancing the expression of the epigenetic enzyme JMJD3 demethylase, which activates IRF4 expression. CCL17 mediated the proinflammatory and algesic actions of GM‐CSF 96 that was independent of T/B cells and TNF/IL‐1β. GM‐CSF also regulates the survival of neutrophils and primes them for the production of reactive oxygen species, thus contributing to neutrophil‐mediated pathological processes including NETosis 99. Based on strong preclinical data, biologically based antagonists of GM‐CSF or its receptor have been developed 100. RA patients have elevated GM‐CSF concentrations in serum and synovial fluid and high expression of GM‐CSF‐R in inflamed synovial tissue 101, 102. Moreover, synovial fluid TNF and IL‐1 further amplify the production GM‐CSF 103.

Several clinical trials were performed to test the efficacy and safety of therapeutic antibody against the GM‐CSF receptor in patients with RA 4. The GM‐CSF‐R is composed of a specific ligand‐binding α‐chain (GM‐CSFRα) and a signal‐transducing β‐chain (GM‐CSFRβ), which transmits the intracellular Janus kinase (JAK)2/STAT‐3/STAT‐5 signalling. Mavrilimumab (CAM‐3001) is a fully human IgG4 monoclonal antibody that binds the GM‐CSF‐R and antagonizes the binding of GM‐CSF 4. Moreover, it internalizes with the GM‐CSF‐R and reduces receptor recycling, thereby reducing the receptor expression on the cell surface. The benefit of blocking the GM‐CSF‐R was demonstrated first by Greven *et al*. 100 in preclinical studies using the collagen‐induced arthritis mouse model. Treatment with anti‐mouse GM‐CSF‐R neutralizing antibody (CAM‐3003) inhibited clinical signs and symptoms of arthritis dose‐dependently and greatly reduced the number of activated macrophages within the inflamed synovium 100. In a phase 1 study of RA patients, mavrilimumab administered intravenously was safe and reduced C‐reactive protein (CRP) and erythrocyte sedimentation rate (ESR) levels 104. The efficacy of mavrilimumab in RA patients was demonstrated in two phase IIa studies 105, 106. In the Environment and Reproductive Health (EARTH) study 106, mavrilimumab modulated the pathophysiological pathways associated with RA, including acute‐phase proteins (CRP and IL‐6) and molecules involved in bone damage [matrix metalloproteinase‐3 (MMP3)], confirming that GM‐CSF acts upstream in the pathogenesis of RA. A subsequent phase IIb trial (EARTH EXPLORER 1), demonstrated that mavrilimumab represented a rapid, effective and well‐tolerated potential treatment for RA patients who had previously failed treatment with conventional disease‐modifying anti‐rheumatic drugs (DMARDs), and even biologicals (anti‐cytokine therapies) targeting other inflammatory pathways 107, and this emphasized an important pathogenic role for the GM‐CSF axis in difficult‐to‐treat RA. Subsequently, results from the EARTH EXPLORER 2 trial confirmed that mavrilimumab was efficacious and well‐tolerated in RA patients who were inadequate responders to both conventional DMARDs and TNF‐inhibitors 108. Moreover, a subanalysis of the EARTH EXPLORER 2 trial, conducted to dissect whether mavrilimumab and golimumab (TNF inhibitor) in both conventional DMARDs and TNF‐inhibitor inadequate responder RA patients may differ in the modulation of peripheral biomarkers and pathophysiological pathways, showed that serum levels of CCL22 and CCL17 were suppressed selectively by mavrilimumab but not golimumab, while CXCL13 and ICAM1 levels were suppressed by golimumab but not mavrilimumab. These findings suggest that these two biological DMARDs act on distinct pathways in patients with RA. This was consistent with animal model data showing that the pathogenic role of GM‐CSF in arthritis was CCL17‐dependent and TNF‐independent 96. Moreover, mavrilimumab was associated with sustained suppression of other inflammatory markers, including vascular endothelial growth factor (VEGF), MMP1 and MMP3, suggesting its role attenuating aberrant tissue angiogenesis and structural damage 109. To date, several monoclonal antibodies blocking GM‐CSF have been developed: lenzilumab (KB003), gimsilumab (MORAb‐022), MOR103 and namilumab (MT203). MOR103, an IgG1 anti‐GM‐CSF 110 tested in a randomized phase Ib/IIa, double‐blind, placebo‐controlled trial in RA patients, showed a better clinical response [in terms of disease activity score in 28 joints (DAS28)] than placebo‐treated patients in a dose‐dependent manner, along with a favourable safety profile 111.Thus, results to date suggest that targeting the GM‐CSF pathway could be a good treatment option in RA, particularly for patients not responding to TNF blockers.

## Improving sustained remission in RA patients: uncovering mediators of remission

The introduction of anti‐cytokine therapies for management of RA (i.e. TNF inhibitors and anti‐IL‐6R antibody) has revolutionized the wellbeing of patients. However, despite this progress 112, a significant number of RA patients do not achieve sustained remission, and this highlights the unmet need for therapies with alternative mechanisms of action to treat RA 9. Moreover, the uncertainty of disease relapse in RA patients in remission after tapering or discontinuation of treatment 113, 114, 115, 116 strongly supports the need for a more comprehensive study of the mediators associated with resolution of inflammation. A recent elegant paper led by Rauber 116 showed that RA patients in remission had increased ILC2 producing IL‐9 in circulation and synovium, and that ILC2/IL‐9 fostered the resolution of inflammation and restored joint immune‐homeostasis in experimental arthritis models 116. Mechanistically, they showed that treatment with IL‐9 promoted ILC2‐dependent regulatory T cell activation that effectively induced resolution of inflammation and protection of bone. These observations suggest that studying mechanisms of remission could provide additional or alternative targets for RA therapy.

## Conclusion

Multiple lines of evidence support the concept that myeloid cells are key regulators driving the innate immune response to support chronic, difficult‐to‐reverse (auto)immune inflammation in RA. Several inflammatory mediators (i.e. TNF and GM‐CSF) lead to epigenetic reprogramming of macrophages that promote chronicity within the synovial tissue. The TNF‐enriched synovial environment in concert with IFN‐α may limit the development of myeloid cells tolerance to TLR stimuli, and thus prevents the development of normal homeostatic immune response. GM‐CSF, also enriched in the synovial environment, profoundly activates myeloid cells by metabolic reprogramming that is critical for an increase production of proinflammatory cytokines following TLR restimulation and release of algesic CCL17. In addition, low expression of anti‐inflammatory regulators, e.g. *AXL* in dendritic cells, driven by a specific epigenetic signature, leads to high endogenous expression of IL‐6 that may contribute to chronicity of inflammation and activation of lymphocytes. Future studies investigating the pathophysiological mechanisms contributing to the perpetuation of inflammation in RA will provide a better understanding of the transition phase from acute to chronic inflammation. In addition, a better understanding of mechanisms of remission could provide alternative ways to promote resolution of chronic inflammation.

## Disclosure

Authors declare no conflicts of interest.
